# *trans*-Dichloridobis(quinoline-κ*N*)palladium(II)

**DOI:** 10.1107/S1600536811055954

**Published:** 2012-01-14

**Authors:** Kwang Ha

**Affiliations:** a School of Applied Chemical Engineering, The Research Institute of Catalysis, Chonnam National University, Gwangju 500-757, Republic of Korea

## Abstract

In the title complex, [PdCl_2_(C_9_H_7_N)_2_], the Pd^II^ ion is four-coordinated in an essentially square-planar environment defined by two N atoms from two quinoline ligands and two Cl^−^ anions. The Pd atom is located on an inversion centre, and thus the asymmetric unit contains one half of the complex; the PdN_2_Cl_2_ unit is exactly planar. The dihedral angle between the PdN_2_Cl_2_ unit and quinoline ligand is 85.63 (8)°. In the crystal, the complex mol­ecules are stacked into columns along the *b* axis. In the columns, several inter­molecular π–π inter­actions between the six-membered rings are present, the shortest ring centroid–centroid distance being 3.764 (3) Å between pyridine rings.

## Related literature

For the crystal structure of the related Pt^II^ complex *cis*-[PtCl_2_(quinoline)_2_]·0.25DMF, see: Davies *et al.* (2001 ▶).
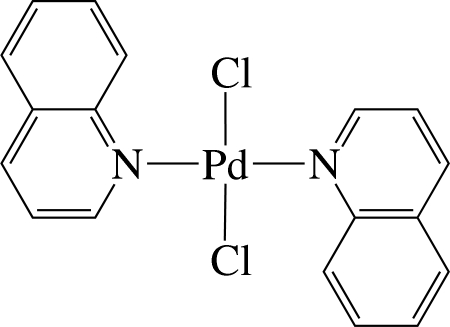


## Experimental

### 

#### Crystal data

[PdCl_2_(C_9_H_7_N)_2_]
*M* *_r_* = 435.61
Monoclinic, 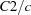

*a* = 16.430 (3) Å
*b* = 7.0050 (11) Å
*c* = 16.118 (2) Å
β = 119.532 (3)°
*V* = 1614.0 (4) Å^3^
*Z* = 4
Mo *K*α radiation
μ = 1.48 mm^−1^
*T* = 200 K
0.31 × 0.13 × 0.11 mm

#### Data collection

Bruker SMART 1000 CCD diffractometer
Absorption correction: multi-scan (*SADABS*; Bruker, 2000 ▶) *T*_min_ = 0.869, *T*_max_ = 1.000
4776 measured reflections
1577 independent reflections
1125 reflections with *I* > 2σ(*I*)
*R*_int_ = 0.041

#### Refinement

*R*[*F* ^2^ > 2σ(*F* ^2^)] = 0.040
*wR*(*F* ^2^) = 0.095
*S* = 1.05
1577 reflections
106 parameters
H-atom parameters constrained
Δρ_max_ = 1.30 e Å^−3^
Δρ_min_ = −0.40 e Å^−3^

### 

Data collection: *SMART* (Bruker, 2000 ▶); cell refinement: *SAINT* (Bruker, 2000 ▶); data reduction: *SAINT*; program(s) used to solve structure: *SHELXS97* (Sheldrick, 2008 ▶); program(s) used to refine structure: *SHELXL97* (Sheldrick, 2008 ▶); molecular graphics: *ORTEP-3* (Farrugia, 1997 ▶) and *PLATON* (Spek, 2009 ▶); software used to prepare material for publication: *SHELXL97*.

## Supplementary Material

Crystal structure: contains datablock(s) global. DOI: 10.1107/S1600536811055954/tk5039sup1.cif

Additional supplementary materials:  crystallographic information; 3D view; checkCIF report

## Figures and Tables

**Table:** 

Pd1—N1	2.035 (4)
Pd1—Cl1	2.2973 (12)

**Table:** 

N1—Pd1—Cl1	89.53 (10)

**Fig. 1.**
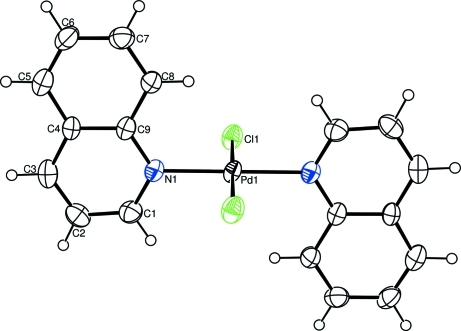
A view of the molecular structure of the title complex, with displacement ellipsoids drawn at the 40% probability level and the atom numbering. Unlabelled atoms are related to the reference atoms by the (-x, 1 - y, -z) symmetry transformation.

**Fig. 2.**
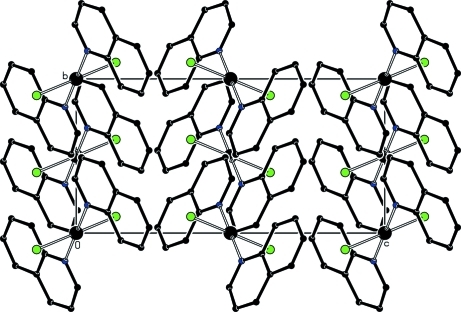
A view of the unit-cell contents of the title complex, along the a axis.

## supplementary crystallographic information

### Comment

In the title complex, [PdCl_2_(quinoline)_2_], the Pd^II^ ion is four-coordinated in an essentially square-planar environment by two N atoms from two quinoline ligands and two Cl^-^ anions (Fig. 1 and Table 1). The Cl atoms are in *trans* conformation with respect to each other. By contrast, in the analogous Pt^II^ complex [PtCl_2_(quinoline)_2_].0.25DMF (DMF = *N,N*-dimethylformamide), the Cl atoms are in *cis* conformation (Davies *et al.*, 2001).

The Pd atom is located on an inversion centre, and thus the asymmetric unit contains one half of the complex; the PdN_2_Cl_2_ unit is exactly planar. The nearly planar quinoline ligands, with a maximum deviation of 0.015 (4) Å from the least-squares plane, are parallel. The dihedral angle between the PdN_2_Cl_2_ unit and quinoline ligand is 85.63 (8)°. The Cl atoms are almost perpendicular to the quinoline planes, with the bond angle <N1—Pd1—Cl1 = 89.53 (10)°. In the crystal, the complex molecules are stacked into columns along the *b* axis (Fig. 2). In the columns, several intermolecular π-π interactions between the six-membered rings are present, the shortest ring centroid-centroid distance being 3.764 (3) Å between pyridyl rings.

### Experimental

To a solution of Na_2_PdCl_4_ (0.2943 g, 1.000 mmol) in H_2_O (20 ml) was added quinoline (0.2590 g, 2.005 mmol). The mixture was stirred for 3 h at room temperature. The formed precipitate was separated by filtration, washed with H_2_O and EtOH, and dried at 50 °C, to give a yellow powder (0.3706 g). Crystals suitable for X-ray analysis were obtained by slow evaporation from its dimethyl sulfoxide (DMSO) solution at 90 °C.

### Refinement

H atoms were positioned geometrically and allowed to ride on their respective parent atoms [C—H = 0.95 Å and *U*_iso_(H) = 1.2*U*_eq_(C)]. The highest peak (1.30 e Å^-3^) and the deepest hole (-0.40 e Å^-3^) in the final difference Fourier map were located 1.01 Å and 1.49 Å from the atoms Pd1 and H5, respectively.

### Crystal data

**Table:**

[PdCl_2_(C_9_H_7_N)_2_]	*F*(000) = 864
*M**_r_* = 435.61	*D*_x_ = 1.793 Mg m^−^^3^
Monoclinic, *C*2/*c*	Mo *K*α radiation, λ = 0.71073 Å
Hall symbol: -C 2yc	Cell parameters from 1841 reflections
*a* = 16.430 (3) Å	θ = 2.9–25.6°
*b* = 7.0050 (11) Å	µ = 1.48 mm^−^^1^
*c* = 16.118 (2) Å	*T* = 200 K
β = 119.532 (3)°	Block, yellow
*V* = 1614.0 (4) Å^3^	0.31 × 0.13 × 0.11 mm
*Z* = 4	

### Data collection

**Table:**

Bruker SMART 1000 CCD diffractometer	1577 independent reflections
Radiation source: fine-focus sealed tube	1125 reflections with *I* > 2σ(*I*)
graphite	*R*_int_ = 0.041
φ and ω scans	θ_max_ = 26.0°, θ_min_ = 2.9°
Absorption correction: multi-scan (*SADABS*; Bruker, 2000)	*h* = −19→20
*T*_min_ = 0.869, *T*_max_ = 1.000	*k* = −8→8
4776 measured reflections	*l* = −18→19

### Refinement

**Table:**

Refinement on *F*^2^	Primary atom site location: structure-invariant direct methods
Least-squares matrix: full	Secondary atom site location: difference Fourier map
*R*[*F*^2^ > 2σ(*F*^2^)] = 0.040	Hydrogen site location: inferred from neighbouring sites
*wR*(*F*^2^) = 0.095	H-atom parameters constrained
*S* = 1.05	*w* = 1/[σ^2^(*F*_o_^2^) + (0.0442*P*)^2^] where *P* = (*F*_o_^2^ + 2*F*_c_^2^)/3
1577 reflections	(Δ/σ)_max_ < 0.001
106 parameters	Δρ_max_ = 1.30 e Å^−^^3^
0 restraints	Δρ_min_ = −0.40 e Å^−^^3^

### Special details

**Table:**

Geometry. All e.s.d.'s (except the e.s.d. in the dihedral angle between two l.s. planes) are estimated using the full covariance matrix. The cell e.s.d.'s are taken into account individually in the estimation of e.s.d.'s in distances, angles and torsion angles; correlations between e.s.d.'s in cell parameters are only used when they are defined by crystal symmetry. An approximate (isotropic) treatment of cell e.s.d.'s is used for estimating e.s.d.'s involving l.s. planes.
Refinement. Refinement of *F*^2^ against ALL reflections. The weighted *R*-factor *wR* and goodness of fit *S* are based on *F*^2^, conventional *R*-factors *R* are based on *F*, with *F* set to zero for negative *F*^2^. The threshold expression of *F*^2^ > σ(*F*^2^) is used only for calculating *R*-factors(gt) *etc*. and is not relevant to the choice of reflections for refinement. *R*-factors based on *F*^2^ are statistically about twice as large as those based on *F*, and *R*- factors based on ALL data will be even larger.

### Fractional atomic coordinates and isotropic or equivalent isotropic displacement parameters (Å^2^)

**Table:**

	*x*	*y*	*z*	*U*_iso_*/*U*_eq_	
Pd1	0.0000	0.5000	0.0000	0.0367 (2)	
Cl1	−0.01202 (8)	0.3930 (2)	0.12827 (8)	0.0488 (3)	
N1	0.1092 (2)	0.3186 (6)	0.0364 (3)	0.0381 (9)	
C1	0.0916 (3)	0.1476 (7)	−0.0041 (3)	0.0451 (12)	
H1	0.0288	0.1172	−0.0498	0.054*	
C2	0.1606 (4)	0.0098 (7)	0.0169 (4)	0.0480 (13)	
H2	0.1449	−0.1106	−0.0142	0.058*	
C3	0.2514 (4)	0.0518 (7)	0.0833 (4)	0.0490 (14)	
H3	0.2996	−0.0392	0.0988	0.059*	
C4	0.2721 (3)	0.2324 (7)	0.1284 (3)	0.0356 (10)	
C5	0.3633 (3)	0.2877 (8)	0.1971 (3)	0.0505 (13)	
H5	0.4134	0.1997	0.2162	0.061*	
C6	0.3806 (4)	0.4638 (8)	0.2362 (4)	0.0519 (14)	
H6	0.4426	0.4991	0.2819	0.062*	
C7	0.3078 (4)	0.5939 (9)	0.2100 (3)	0.0496 (13)	
H7	0.3211	0.7172	0.2383	0.060*	
C8	0.2180 (3)	0.5483 (7)	0.1448 (3)	0.0421 (12)	
H8	0.1692	0.6384	0.1283	0.051*	
C9	0.1979 (3)	0.3651 (7)	0.1018 (3)	0.0373 (11)	

### Atomic displacement parameters (Å^2^)

**Table:**

	*U*^11^	*U*^22^	*U*^33^	*U*^12^	*U*^13^	*U*^23^
Pd1	0.0248 (3)	0.0445 (3)	0.0377 (3)	0.0052 (2)	0.0129 (2)	0.0054 (2)
Cl1	0.0436 (7)	0.0605 (9)	0.0453 (7)	0.0101 (7)	0.0243 (6)	0.0128 (6)
N1	0.030 (2)	0.041 (2)	0.042 (2)	0.0018 (18)	0.0168 (18)	0.0036 (19)
C1	0.040 (3)	0.046 (3)	0.050 (3)	−0.008 (2)	0.023 (2)	−0.002 (2)
C2	0.065 (4)	0.033 (3)	0.054 (3)	−0.003 (3)	0.035 (3)	0.000 (2)
C3	0.048 (3)	0.047 (3)	0.061 (3)	0.014 (2)	0.033 (3)	0.016 (3)
C4	0.032 (2)	0.039 (3)	0.040 (3)	0.007 (2)	0.020 (2)	0.008 (2)
C5	0.037 (3)	0.062 (4)	0.050 (3)	0.010 (3)	0.019 (2)	0.011 (3)
C6	0.032 (3)	0.071 (4)	0.044 (3)	−0.001 (3)	0.012 (2)	−0.001 (3)
C7	0.046 (3)	0.060 (3)	0.041 (3)	−0.005 (3)	0.021 (2)	−0.008 (3)
C8	0.035 (3)	0.042 (3)	0.046 (3)	0.001 (2)	0.018 (2)	−0.002 (2)
C9	0.033 (3)	0.043 (3)	0.037 (3)	0.005 (2)	0.018 (2)	0.009 (2)

### Geometric parameters (Å, °)

**Table:**

Pd1—N1	2.035 (4)	C3—H3	0.9500
Pd1—N1^i^	2.035 (4)	C4—C5	1.409 (6)
Pd1—Cl1	2.2973 (12)	C4—C9	1.421 (6)
Pd1—Cl1^i^	2.2973 (12)	C5—C6	1.350 (7)
N1—C1	1.326 (6)	C5—H5	0.9500
N1—C9	1.351 (5)	C6—C7	1.393 (8)
C1—C2	1.397 (7)	C6—H6	0.9500
C1—H1	0.9500	C7—C8	1.362 (7)
C2—C3	1.373 (7)	C7—H7	0.9500
C2—H2	0.9500	C8—C9	1.418 (7)
C3—C4	1.414 (6)	C8—H8	0.9500
			
N1—Pd1—N1^i^	180.0 (2)	C5—C4—C3	122.9 (4)
N1—Pd1—Cl1	89.53 (10)	C5—C4—C9	118.6 (5)
N1^i^—Pd1—Cl1	90.47 (10)	C3—C4—C9	118.4 (4)
N1—Pd1—Cl1^i^	90.47 (10)	C6—C5—C4	121.0 (5)
N1^i^—Pd1—Cl1^i^	89.53 (10)	C6—C5—H5	119.5
Cl1—Pd1—Cl1^i^	180.00 (9)	C4—C5—H5	119.5
C1—N1—C9	119.4 (4)	C5—C6—C7	120.3 (5)
C1—N1—Pd1	118.3 (3)	C5—C6—H6	119.8
C9—N1—Pd1	122.2 (3)	C7—C6—H6	119.8
N1—C1—C2	123.4 (5)	C8—C7—C6	121.4 (5)
N1—C1—H1	118.3	C8—C7—H7	119.3
C2—C1—H1	118.3	C6—C7—H7	119.3
C3—C2—C1	118.7 (5)	C7—C8—C9	119.5 (5)
C3—C2—H2	120.6	C7—C8—H8	120.3
C1—C2—H2	120.6	C9—C8—H8	120.3
C2—C3—C4	119.1 (5)	N1—C9—C8	120.1 (4)
C2—C3—H3	120.4	N1—C9—C4	120.9 (4)
C4—C3—H3	120.4	C8—C9—C4	119.1 (4)
			
Cl1—Pd1—N1—C1	93.7 (3)	C5—C6—C7—C8	−0.1 (8)
Cl1^i^—Pd1—N1—C1	−86.3 (3)	C6—C7—C8—C9	−0.6 (8)
Cl1—Pd1—N1—C9	−84.5 (3)	C1—N1—C9—C8	179.2 (4)
Cl1^i^—Pd1—N1—C9	95.5 (3)	Pd1—N1—C9—C8	−2.6 (6)
C9—N1—C1—C2	−0.2 (7)	C1—N1—C9—C4	−0.7 (6)
Pd1—N1—C1—C2	−178.5 (3)	Pd1—N1—C9—C4	177.5 (3)
N1—C1—C2—C3	0.5 (7)	C7—C8—C9—N1	−179.4 (4)
C1—C2—C3—C4	0.2 (7)	C7—C8—C9—C4	0.5 (7)
C2—C3—C4—C5	−179.8 (5)	C5—C4—C9—N1	−179.9 (4)
C2—C3—C4—C9	−1.1 (7)	C3—C4—C9—N1	1.4 (6)
C3—C4—C5—C6	177.8 (5)	C5—C4—C9—C8	0.2 (6)
C9—C4—C5—C6	−0.9 (7)	C3—C4—C9—C8	−178.6 (4)
C4—C5—C6—C7	0.8 (8)		

Symmetry codes: (i) −*x*, −*y*+1, −*z*.
